# A New Rabbit-Skin Model to Evaluate Protective Efficacy of Tuberculosis Vaccines

**DOI:** 10.3389/fmicb.2017.00842

**Published:** 2017-05-17

**Authors:** Huiyu Chen, Xun Liu, Xingming Ma, Qian Wang, Guang Yang, Hongxia Niu, Shuaixiang Li, Bingzheng He, Shanshan He, Arthur M. Dannenberg, Bingdong Zhu, Ying Zhang

**Affiliations:** ^1^Department of Immunology, School of Basic Medical Sciences, Lanzhou UniversityLanzhou, China; ^2^Gansu Key Lab of Evidence Based Medicine and Clinical Transfer Medicine & Lanzhou Center for Tuberculosis Research, Lanzhou UniversityLanzhou, China; ^3^School of Basic Medical Sciences, Institute of Pathogen Biology, Lanzhou UniversityLanzhou, China; ^4^School of Basic Medical Sciences, Institute of Chinese Integrative Medicine, Lanzhou UniversityLanzhou, China; ^5^Departments of Environmental Health Sciences, Epidemiology, Molecular Microbiology and Immunology, and Pathology, Johns Hopkins Medical InstitutionsBaltimore, MD, United States; ^6^Department of Molecular Microbiology and Immunology, Bloomberg School of Public Health, Johns Hopkins UniversityBaltimore, MD, United States

**Keywords:** tuberculosis, subunit vaccine, BCG, rabbit skin model, pathology

## Abstract

**Background:** BCG protection is suboptimal and there is significant interest to develop new tuberculosis (TB) vaccines. However, there are significant limitations of the current vaccine evaluation systems in the mouse model. Here, we developed a BCG-challenge rabbit skin model as a new way to evaluate the protective efficacy of selected TB subunit vaccine candidates.

**Methods:** Rabbits were immunized with subunit vaccines, including EAMM (ESAT6-Ag85B-MPT64_<190−198>_-Mtb8.4), MH (Mtb10.4-HspX), and LT70 (ESAT6-Ag85B-MPT64_<190−198>_-Mtb8.4-Rv2626c) three times subcutaneously every 3-weeks and challenged with the attenuated *Mycobacterium bovis* BCG intradermally 6-weeks after last immunization. The immune response induced by the vaccine candidates was measured, the histopathology induced by the BCG challenge was studied, and the number of bacilli in the liquefied caseum was determined.

**Results:** The subunit vaccines generated high antigen-specific IgG antibodies and fastened the liquefaction and healing process, and significantly reduced the viable BCG load. The subunit vaccine LT70 and EAMM-MH reduced BCG bacterial load in comparison to proteins EAMM, MH, Rv2626c, and also BCG itself. The Koch phenomena induced by the LT70 and combination of EAMM-MH were the same as that produced by BCG itself and were more rapid than those induced by the other proteins and the saline controls.

**Conclusions:** The subunit vaccines LT70 and the combination of EAMM-MH showed promising protective efficacy as expected in the rabbit skin model, which can serve as a visual and convenient new model for evaluating TB vaccines.

## Introduction

Tuberculosis (TB) remains a major threat to global public health. About one-third of the world's population is infected with *Mycobacterium tuberculosis* (*M. tuberculosis*) (Lonnroth et al., 2010). Drug-resistant TB and co-infection with human immunodeficiency virus (HIV) make TB more difficult to control (Shah et al., 2007; Ghebreyesus et al., 2010). The current TB vaccine BCG has a high efficacy in preventing infants and children from miliary and meningeal tuberculosis (Colditz et al., 1994; Trunz et al., 2006), but shows variable efficacy against pulmonary tuberculosis (ranging from 0 to 80%) in adults (Fine, 1995). In the last decade, numerous novel TB vaccine candidates (including subunit vaccines) have been developed (Brennan and Thole, 2012; Wang et al., 2013). The subunit vaccines are designed by delivering one or more potentially protective mycobacterial antigens with an adjuvant or a viral vector. In some experiments, subunit vaccines have shown comparable or even better protective efficacy than BCG (Lindenstrom et al., 2009).

Animal models are essential to early stages of vaccine evaluation (Meyer and McShane, 2013). Among them, non-human primates have been used, because their genomes, physiology, and immune systems are remarkably similar to those of human beings (Flynn et al., 2003). Cattle are the natural host for *Mycobacterium bovis* and most likely to be infected with *M. bovis* through inhalation of aerosolized droplets, which produce strikingly similar immune responses (Pollock et al., 2006). However, both non-human primates and cattle are large animals and the containment facilities are costly and not easy to evaluate different vaccine candidates. Guinea pigs and mice are the most commonly used animal models in which to study TB (Young, 2009). However, guinea pigs produce weak cell-mediated immunity (CMI), and mice produce weak delayed-type hypersensitivity (DTH; Helke et al., 2006). For these reasons, mice and guinea pigs rarely produce liquefied lesions and pulmonary cavities, which play an important role in exacerbation and transmission of human *M. tuberculosis* infection (Helke et al., 2006). Rabbits are superior to guinea pigs and mice as an animal model because of their ability to form liquefied and cavitary lesions which are similar to those found in human beings (Dannenberg, 2001, 2009, 2010; Helke et al., 2006).

Previously, we described a rabbit skin liquefaction model (Zhang et al., 2010; Sun et al., 2012). This model provides a more visual and convenient method to study TB pathogenesis by injecting rabbits intradermally with live BCG. Liquefied and ulcerative lesions could be produced, which were comparable to those in the lung even though skin and lungs are different tissues (Minassian et al., 2011). We have previously used the rabbit skin model to evaluate the virulence of different mycobacteria (Zhang et al., 2010) and the effects of immunomodulators on liquefaction and ulceration (Sun et al., 2012).

In the present study, we adapted the rabbit skin liquefaction model to evaluate the efficacy of TB subunit vaccines. The vaccines tested were fusion proteins EAMM (ESAT6-Ag85B-MPT64_<190−198>_-Mtb8.4), MH (Mtb10.4-HspX), and LT70 (ESAT6-Ag85B-MPT64_<190−198>_-Mtb8.4-Rv2626c), which consist of antigens expressed at different metabolic stages of *M. tuberculosis*, such as ESAT6 (Ulrichs et al., 1998), MPT64_190−198_ (Silver et al., 1995), Ag85B (Lozes et al., 1997), Mtb8.4 (Coler et al., 1998), Mtb10.4 (Skjot et al., 2000), which are mainly expressed by replicating bacteria, whereas HspX and Rv2626c are highly expressed in dormant bacteria. In previous studies, the subunit vaccines LT70, EAMM, and combination of EAMM-MH were effective in protection against virulent *M. tuberculosis* infection in the mouse model (Xin et al., 2013; Liu et al., 2016). In this study, we found that the subunit vaccines LT70 and EAMM-MH also showed promising protective efficacy in the rabbit skin model, which indicates the rabbit skin model is a convenient and more visual model to evaluate TB vaccine candidates.

## Materials and methods

### Mycobacteria, fusion proteins, and adjuvants

BCG Shanghai strain was obtained from Lanzhou Institute of Biological Products and cultured in Middle brook 7H9 liquid medium (BD, NJ, USA) enriched with OADC (BD, NJ, USA). Log phase bacilli were harvested, washed, and adjusted in PBS to a concentration of 5 × 10^7^ CFU/ml.

The fusion proteins EAMM (ESAT6-Ag85B-MPT64_<190−198>_-Mtb8.4), MH (Mtb10.4-HspX), and LT70 (ESAT6-Ag85B-MPT64_<190−198>_-Mtb8.4-Rv2626c) were overexpressed in *E. coli* strain BL21. They were purified by chromatography as previously reported (Xin et al., 2013; Liu et al., 2016). The fusion proteins were mixed with adjuvants N,N′-dimethyl-N,N′-dioctadecylammonium bromide (DDA, Sigma-Aldrich, Poole, UK) and poly(I:C) (Sigma-Aldrich, Poole, UK) to construct the subunit vaccines.

### Animals

Female New Zealand white rabbits weighing 2.0–3.0 kg were provided by Lanzhou Veterinary Research Institute (Chinese Academy of Agricultural Sciences). Animals were housed under conditions with *ad libitum* access to chow and water in Animal Center of Lanzhou University. These animal experiments were approved by the Animal Care and Use Committee of Lanzhou University [permit number: SYX(Gan)2013-001].

### Vaccinations

Two experiments were performed separately to observe the utility of the rabbit skin model in TB vaccine evaluation. First, EAMM, MH, and EAMM-MH were evaluated. Rabbits were vaccinated three times at 3-week intervals with the subunit vaccines and injected with EAMM 20 μg, MH 20 μg, MH 10 μg plus EAMM 10 μg, respectively, in 200 μl solution containing adjuvant of DDA 250 μg and poly(I:C) 50 μg. In addition, a group received BCG as 5 × 10^6^ CFU resuspended in 200 μl saline at the time of the first subunit vaccination and another group received the same volume of saline as the control. There were three rabbits in each group and both flanks were used. All the rabbits were injected subcutaneously.

In order to verify utility of the rabbit skin model for evaluating TB vaccines, we performed the second trial to evaluate EAMM, EAMM-MH, Rv2626c, and LT70, which were shown to have protective effect in our previous studies (Liu et al., 2016). Rabbits were vaccinated three times at 3-week intervals with the subunit vaccines and injected with Rv2626c 10 μg, MH 20 μg, EAMM 20 μg, MH 10 μg plus EAMM 10 μg, and LT70 10 μg, respectively, in 200 μl of a solution containing 250 μg of adjuvant DDA and 50 μg of poly(I:C). In addition, a group received 5 × 10^6^ CFU of BCG resuspended in 200 μl of saline at the time of the first subunit vaccination and another group received the same volume of saline as the control. There were three rabbits in each group.

### Enzyme-linked immunosorbent assay (ELISA) for antibody detection

Following immunization of MH and EAMM, Enzyme-linked immunosorbent assay was performed 6-weeks after the final immunization to determine the serum levels of anti-ESAT6, anti-Ag85B, and anti-HspX. First, the 96-well microtiter plates were coated overnight at 4°C, respectively, with ESAT6, Ag85B, HspX (5 μg/ml antigen in coating buffer 100 μl/well). Then the plates were washed five times with PBS containing 0.05% Tween 20 (PBST). The serum samples were diluted in halving concentrations with PBS from 1:100 to 1:204,800 and applied to the plates. Peroxidase-conjugated goat anti-rabbit IgG (Rockland Immunochemicals Inc., Rockland, ME, USA) was diluted to 1:5,000 and used at 200 μl/well. The plates were incubated at 37°C for 1 h and washed by PBST. Then the colored substrate tetramethylbenzidine (TMB) was added to the plates. After 15 min, stop buffer was added. Serum from rabbits vaccinated with saline was used as the negative control. The cutoff value was double of negative control or 0.100 at OD 450 nm. Sera at OD 450 nm above the cutoff point were considered positive.

### BCG challenge and measurement of the rabbit skin lesions

Rabbits were injected intradermally at two sites on each flank with 100 μl BCG containing 5 × 10^6^ CFU at 6-weeks after last vaccination (**Figure 4A**). The two sites were 2–3 cm apart. The size of the skin lesions (the length multiplied by the width and the thickness; Chandrasekhar et al., 1971; Dannenberg et al., 1972) was measured daily with calipers. The observer was blind to the group identities. In addition, the lesions and normal skin tissues nearby were collected, and the tissue sections were prepared and stained with hematoxylin and eosin. The histopathology was examined.

### Bacterial loads

At the peak stage of liquefaction, the liquefied caseum was collected, weighed, diluted with 1 ml saline, homogenized and cultured on the Middlebrook 7H10 agar enriched with 10% OADC and ampicillin (0.01 mg/ml) (to prevent the growth of contaminating bacteria). After 21 days, the colony-forming units (CFUs) were enumerated. Ziehl-Neelsen acid-fast staining was used to confirm that the bacterial colonies were tubercle bacilli.

### Statistical analysis

The SPSS19.0 software was used to calculate and analyze the means and their standard errors (SE). The sizes of the skin lesions were compared using analysis of variance (ANOVA) (including multivariate and repeated-measurement comparisons). ANOVA and the independent sample *t*-test were used to analyze the antibody titers and bacterial loads. *P* < 0.05 was considered statistically significant.

## Results

### BCG could induce rabbit skin lesions

**Figure 1:**
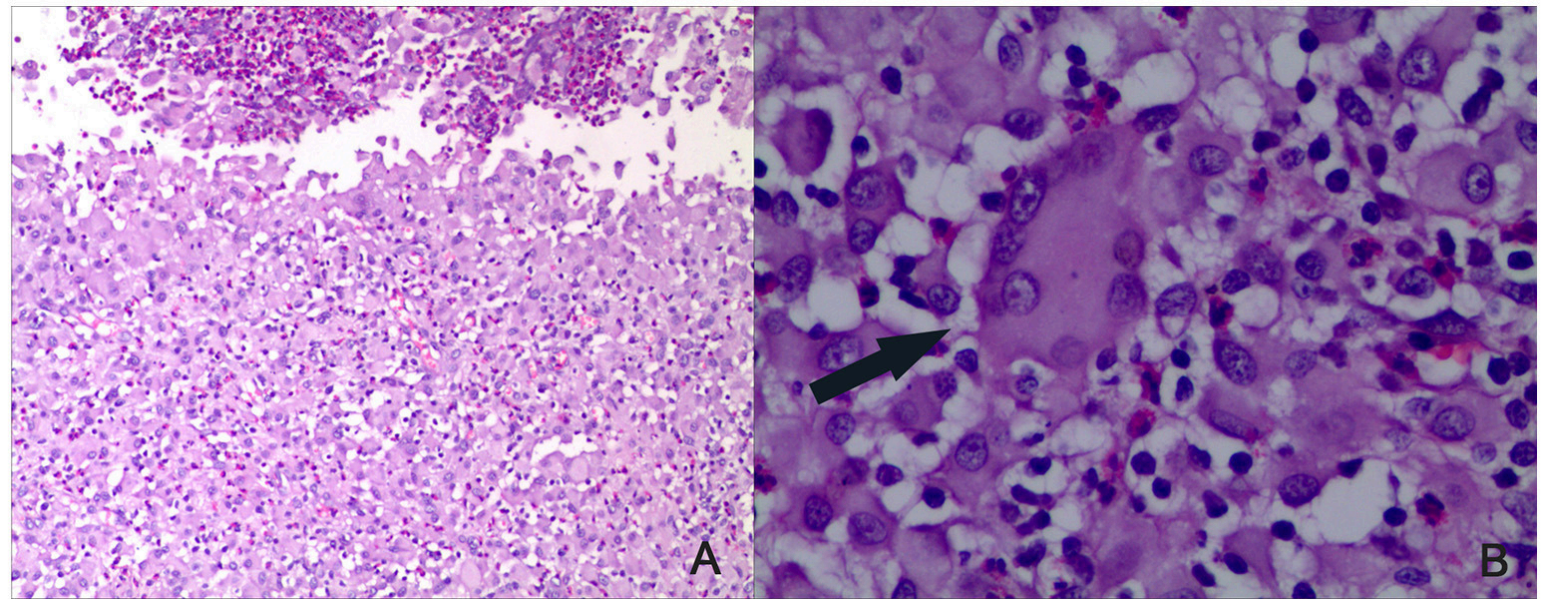
**Histopathology of the rabbit skin lesions (HE staining)**. BCG was used to induce rabbit skin lesion. As all groups showed same pathologic reaction, a representative skin lesions is shown after BCG challenge. After the lesions healed, tissue sections were made. **(A)** Infiltration of epithelioid cells, macrophages, and lymphocytes could be seen along with unabsorbed liquefied material and necrosis (×100). **(B)** Langhans giant cells as indicated by the arrow could be seen (×400).

Intradermal BCG is known to induce healing tuberculous lesions in the rabbit (Zhang et al., 2010). Here, we studied their histopathology and measured the bacterial growth within them. The bacteria in the liquefied caseum were cultured on Middlebrook 7H10 plates and found to be acid-fast. The skin lesions contained epithelioid cells, macrophages, and lymphocytes. Langhans giant cells were present along with unabsorbed liquefied material and necrosis (Figure 1). These findings showed that BCG could cause significant lesions typical of tuberculosis and were not induced by contaminating bacteria, and that BCG could serve as a challenge organism for vaccine evaluation

### Antigen-specific antibodies induced by vaccination

**Figure 2:**
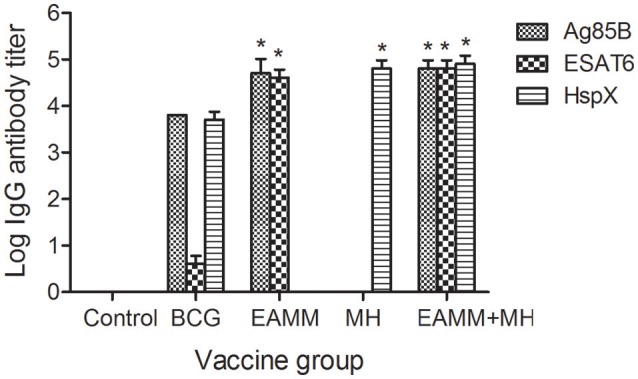
**Titers of antigen-specific IgG antibodies in the vaccinated groups**. About 6-weeks after last vaccination, sera from all three rabbits in each group were obtained and the titers of ESAT6-specific, Ag85B-specific, and HspX-specific IgG antibodies were determined. Each column depicts the corresponding antigen-specific IgG antibody titer. ^*^P < 0.01, relative to saline control and BCG groups.

ELISA was performed 6-weeks after the final immunization to determine the serum levels of antibodies against ESAT6, Ag85B, and HspX. There were no antigen-specific antibodies present in the saline control group. The ESAT6-specific and Ag85B-specific IgG antibody titers in the EAMM group, and HspX-specific IgG antibody titers in the MH group were higher than those in the BCG group. Animals receiving the EAMM-MH subunit vaccine also generated higher levels of ESAT6-specific, Ag85B-specific, and HspX-specific IgG antibodies than the BCG group (Figure 2).

### Effects of different vaccine candidates on the development of rabbit skin lesions following BCG challenge

In our previous study (Sun et al., 2012), we described six stages in the development of tuberculous skin lesions induced by BCG: (a) Granuloma, a solid lesion with no evidence of liquefaction; (b) Onset of liquefaction, a softened lesion with some exudate; (c) Ulcerated ruptured lesion discharging some liquefied caseum; (d) Peak liquefied lesion with lots of liquefied caseum being discharged; (e) Early healing lesion which regressed visibly (the shrinkage had reached 60%) and scabbed; (f) Late healing lesions (the lesions had shrunk more than 75%) and were epithelialized.

**Figure 3:**
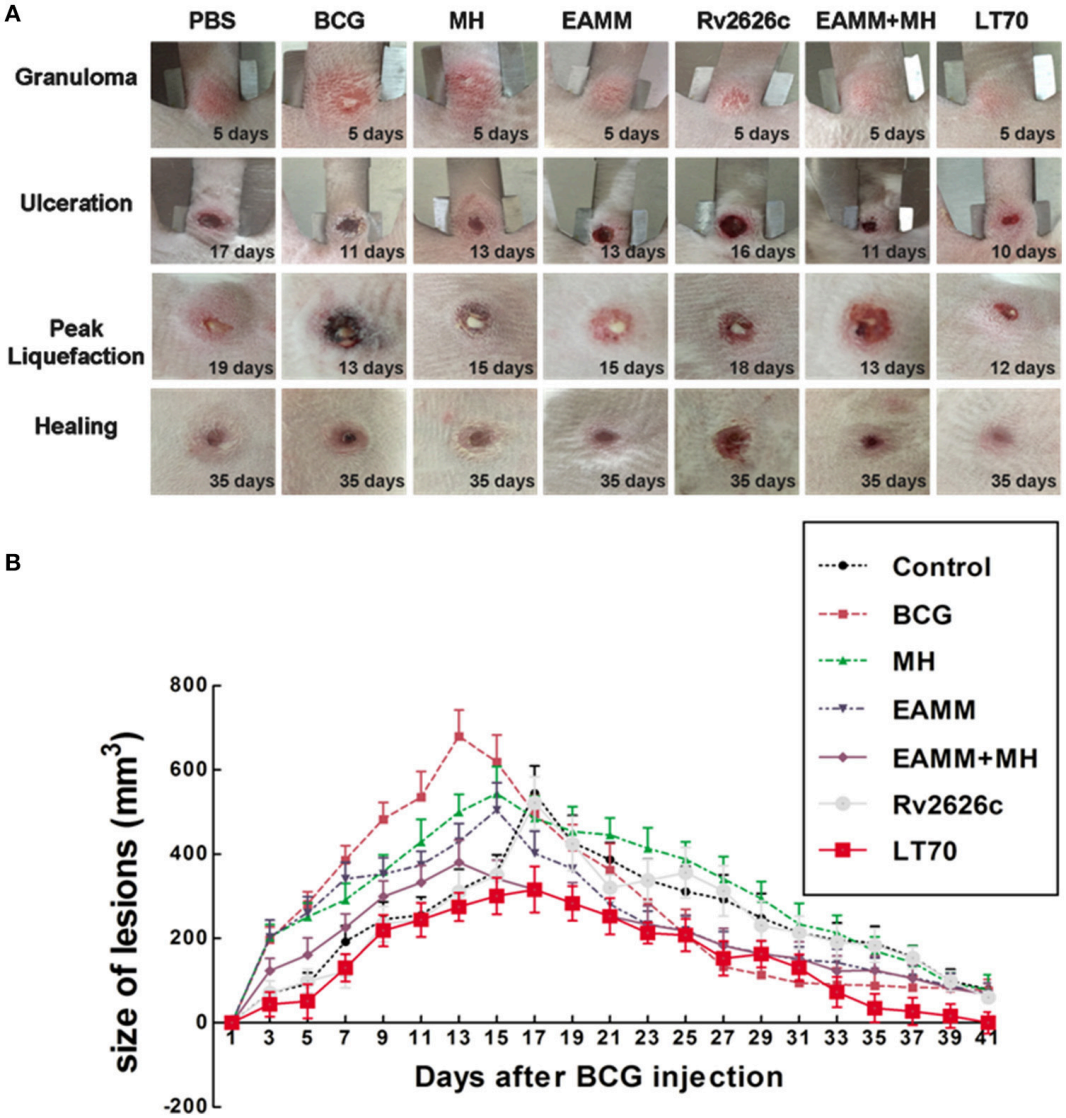
**Volumes of dermal BCG lesions in the different vaccinated groups**. The rabbits in each group were injected intradermally with 5 × 10^6^ CFU of live BCG (100 μl) on each rabbit flank. The lesions were measured daily **(A)**. The lesion size was calculated from their width, length and thickness **(B)**. Each point represents the mean and its standard error. Two representative lesions were chosen from each rabbit.

We assessed the effects of different vaccine candidates on BCG-induced lesions by monitoring the time of the onset of liquefaction, ulceration, liquefaction peak and onset of healing following the BCG challenge (Table 1). The maximum size of the lesion was at the time of the liquefaction peak (Table 1 and Figure 3). In MH and EAMM protein vaccination groups, the lesions reached their maximum sizes at day 15; Group BCG and group EAMM-MH reached the liquefaction peak earlier (at day 13) than the single vaccine groups; Group LT70 reached the liquefaction peak at the earliest time (at day 12) among all groups, while the saline control group (at day 19) was the last to reach the liquefaction peak (Figure 3A). The lesions in the BCG group were the largest, while the lesions in the LT70 and combined EAMM-MH vaccine groups were smaller than the other groups. The volumes of lesions in the saline control and other protein groups were between those of the BCG and LT70 protein groups. The lesions in the LT70 group healed the fastest among all groups, and BCG group and EAMM-MH group healed faster than MH, EAMM, Rv2626c, and saline control groups (Figure 3B). These observations indicate that the BCG and the subunit vaccines accelerated both the liquefaction and the healing processes and that the LT70 was the most effective among them.

**Table 1:** **Effects of different vaccine candidates on BCG-induced rabbit skin lesions**.

	**Time of onset of liquefaction (days post-infection)**	**Time of ulceration (days post-infection)**	**Time of peak of liquefaction (days post-infection)**	**Lesion volumes when liquefaction peaked (mm^3^) (mean ±*SD*)**	**Onset of healing (days post-infection)**
Control	14	17	19	544.78 ± 64.89	31
BCG	8	11	13	679.66 ± 62.31	25
Rv2626c	13	16	18	521.78 ± 61.90	29
MH	10	13	15	544.05 ± 63.60	30
EAMM	10	13	15	504.35 ± 65.46	27
EAMM-MH	8	11	13	380.15 ± 53.84^**^	25
LT70	7	10	12	301.16 ± 40.50^**^	22

** *P < 0.05 vs. Saline and BCG*.

### Effects of vaccine candidates on the bacterial load in the liquefied caseum

**Figure 4:**
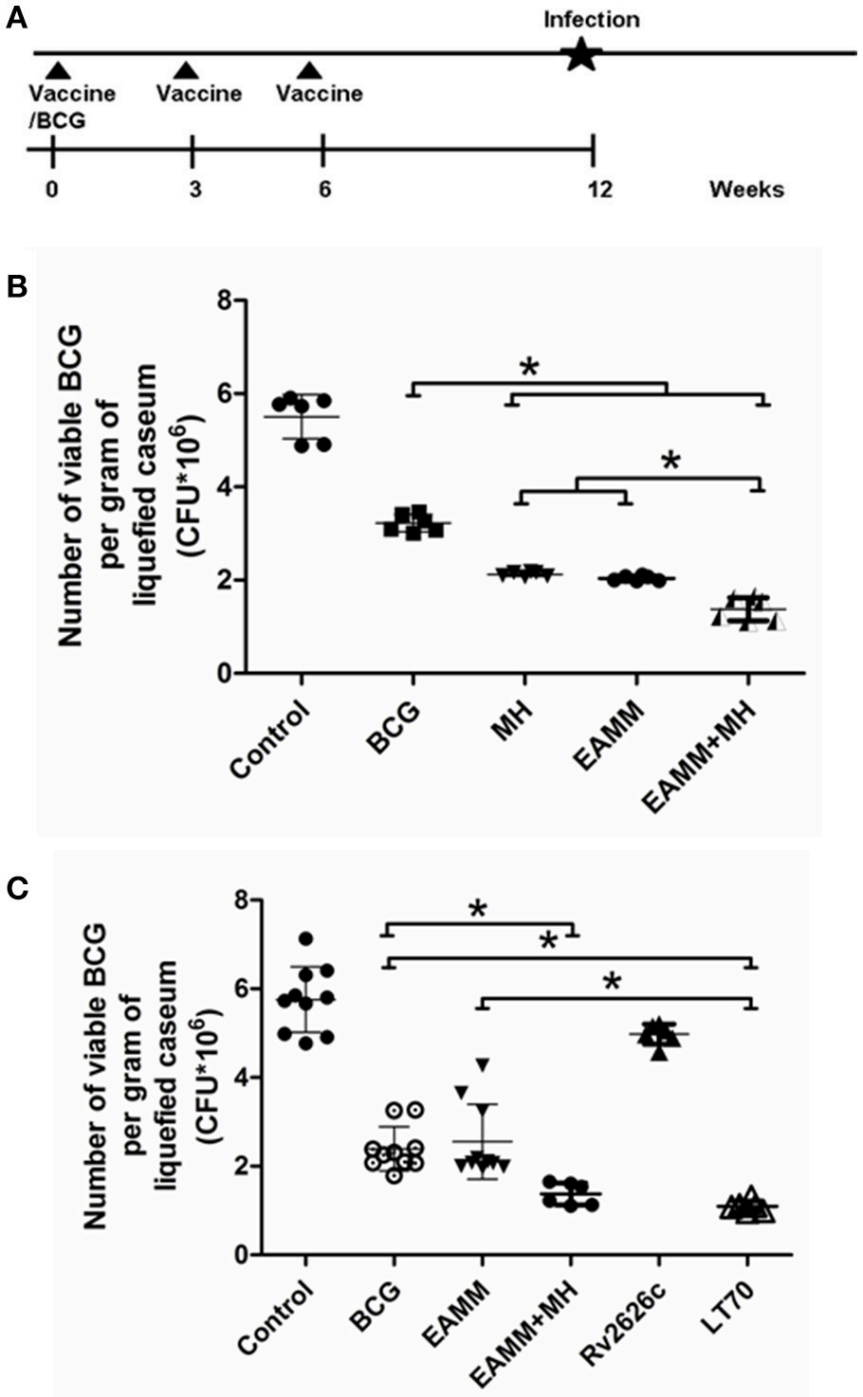
**The number of viable BCG per gram of liquefied caseum at the time the liquefaction peaked**. The liquefied caseum of the rabbit skin lesions was obtained at the time when the liquefaction peaked and the number of bacilli per gram of liquefied material was determined. The schematic for the vaccine evaluation is shown in **(A)**. In the first vaccination trial, we evaluated BCG and the vaccine candidates EAMM, MH, EAMM-MH **(B)**. Then, we evaluated BCG, Rv2626c, EAMM, EAMM-MH, and LT70 in the second vaccination trial **(C)**. The means and their standard errors are shown. ^*^P < 0.05.

The liquefied caseum at the peak of liquefaction was collected, and the CFU count was determined. In the first vaccination trials, we evaluated the vaccines BCG and EAMM, MH, EAMM-MH. The results showed CFU in the BCG group was only lower than that in the saline control group. The bacterial loads in EAMM combined with MH group were the lowest (Figure 4B). Then, we evaluated the vaccines BCG, Rv2626c, EAMM, EAMM-MH, and LT70 in the second vaccination trial. In this experiment, BCG, EAMM-MH, and LT70 groups showed better protective effect than Rv2626c and EAMM did, while LT70 group showed the best effect to control bacterial growth or replication among all groups, and its bacterial load is lower than EAMM and BCG group (*p* < 0.05; Figure 4C). Therefore, LT70 and EAMM-MH showed more effective protection against mycobacterial infection than BCG and the single antigens in the rabbit skin model. Same tendency was found in the mice model challenged with virulent *M. tuberculosis* H37Rv by respiratory way (Xin et al., 2013; Liu et al., 2016).

## Discussion

In our present study, we used the rabbit-skin model to evaluate the protective efficacy of subunit vaccines. The rabbits were immunized with BCG and the subunit vaccines EAMM, MH, EAMM-MH, Rv2626c, and LT70 and challenged with BCG intradermally. Following BCG—challenging, the vaccines accelerated liquefaction and healing and reduced the bacterial load. BCG, EAMM-MH, and LT70 vaccines showed better protective effect compared to the others, while LT70 was the most effective.

The rabbit is an excellent model for human TB, because rabbits and humans readily form liquefied lesions and cavities (Dannenberg, 2001, 2009). Liquefaction is a key process in tuberculosis. It leads to cavity formation that spreads the disease to others. Although mice produce strong CMI, their lesions rarely liquefy. Guinea pigs are susceptible to *M. tuberculosis* and only sometimes develop liquefied lesions and form cavities (Helke et al., 2006). Both mouse and guinea pig models could not completely reflect the real protective efficacy of human beings, because of different susceptibility, and lesion characteristics. In rabbits, during infection with tubercle bacilli CMI and tissue-damaging DTH develops as in human beings, causing granulomas, liquefaction, and cavities successively (Dannenberg, 2009; Zhang et al., 2010; Sun et al., 2012). Consistent with the strength of immune responses, LT70, EAMM-MH, and BCG vaccination induced the most rapid liquefaction and healing of the lesions (Figure 3). LT70 provided the best protection, followed by EAMM-MH, and BCG in the rabbit skin model (Figure 4C), which is the same as in the more tedious mouse model challenged with virulent *M. tuberculosis* (Liu et al., 2016). The greater immune responses mediated by LT70 led to the most rapid ulceration, the most rapid discharge of the bacilli, and the most rapid healing in the rabbit skin model.

Our experiments evaluating vaccine efficacy in rabbit skin showed that the tuberculosis subunit vaccines accelerated the liquefaction and healing processes in TB lesions, and reduced the bacterial load in the liquefied caseum as in the Koch phenomenon. The pathological process is faster when re-infected with BCG because of the immunity developed by first infection. We found that effective vaccines, especially LT70, accelerated the liquefaction and healing processes, and provided some protection against the BCG challenge. In the human skin challenge model for mycobacterium tuberculosis using BCG, the skin biopsy specimens reflected a degree of mycobacterial immunity in previously BCG-vaccinated individuals, which are consistent with our results (Minassian et al., 2012).

The subunit vaccine LT70 and EAMM-MH groups provided the most effective protection, because they had the lowest bacterial load and least lesions or pathology. This effect is consistent with our findings in the mouse model (Liu et al., 2016). We believe that multistage antigens consisting of antigens from both growing and non-growing dormant phase could improve the protective efficacy of subunit vaccines, because these antigens are expressed at different stages of bacterial growth (Aagaard et al., 2011; Liu et al., 2016). The antigens ESAT6 and HspX have been reported to be immunogenic proteins that enhance capacity of BCG to activate human dendritic cells (DCs), promote production of cytokines such as IL-12, and activate CD4^+^ lymphocytes and NK cells. Rv2626c, a latency antigen, that can bind to the surface of murine macrophages and significantly induce the production of tumor necrosis factor (TNF)-α upon stimulation of murine macrophages (Bashir et al., 2010).

Due to biosafety facility limitations we challenged rabbit skin with BCG as a surrogate of virulent *M. tuberculosis*. BCG has previously been shown to develop lesions and liquefaction similar to that produced by a virulent TB strain (Zhang et al., 2010). Thus, in this study, rabbits were immunized subcutaneously with vaccine candidates (including subunit vaccines and BCG) and were challenged with BCG intradermally. We found that the subunit vaccine LT70 and EAMM-MH reduced BCG bacterial load in comparison to proteins EAMM, MH, Rv2626c, and also BCG itself. Nevertheless, we were able to obtain the same finding with regard to LT70 and EAMM-MH vaccine candidates in both the mouse model and in the rabbit skin model here in this study. Thus, the skin model is visual and conveniently observed although it differs from pulmonary TB infection. Moreover, its effect has been confirmed in human skin test (Minassian et al., 2012). The main caveat about using BCG to evaluate new TB vaccines is that BCG is deficient in some virulence factors. However, BCG still keeps some of the virulence of tubercle bacilli and has been used successfully in models to screen potential tuberculosis vaccines (Dannenberg, 1998; Dheda et al., 2010; Minassian et al., 2012). Future studies are needed to determine if BCG can replace *M. tuberculosis* as a challenge for vaccine evaluation in the skin model. This would greatly alleviate the biosafety concerns of working with virulent *M. tuberculosis* besides providing a simple and more visual approach to vaccine evaluation. In this study, we used saline as a control for vaccine evaluation as a vaccien free control, which was also used previously by us and also others in published studies on vaccine evaluation in mice. However, adjuvant may be a more appropriate control and will be used in future studies. Another limitation is that we were only able to evaluate limited antibody response but not cell-mediated immune (CMI) response in the rabbit model as reagents are not available. However, based on our previous studies with the mouse model which we have detected the CMI responses of the fusion protein-based vaccines (Zhang et al., 2010) we would expect to have similar antibody and CMI responses in the rabbit model.

In summary, the rabbit-skin model using BCG challenge is a convenient and novel method of evaluating new TB vaccines. It would be advisable to test all new vaccines with the rabbit-skin model and the rabbit tubercle-count method (Dannenberg, 1998) with virulent human-type tubercle bacilli for the challenge in future studies. Our experiments suggest that it is necessary to use more than one animal model and different routes of infection to evaluate protective efficacy of vaccines (Dheda et al., 2010). Further, studies are needed to assess the utility of the rabbit skin model as a rapid and visual model for TB vaccine development.

## Author contributions

YZ, BZ, and AD designed the experiments; XL, HC, QW, GY, SL, and BH carried out the animal experiments; HN and SH purified the antigens which used as vaccines; XM and XL analyzed sequencing data; HC, XL, and BZ wrote the manuscript.

### Conflict of interest statement

The authors declare that the research was conducted in the absence of any commercial or financial relationships that could be construed as a potential conflict of interest.
